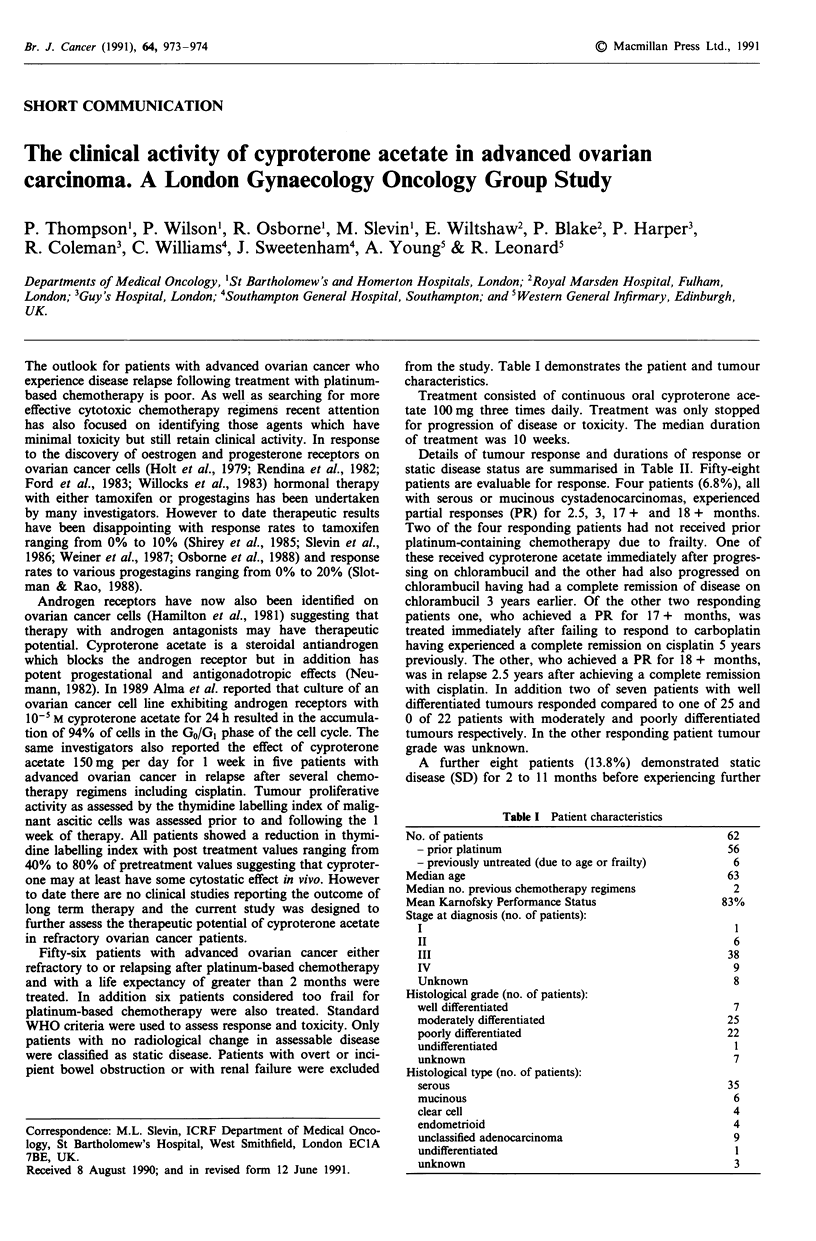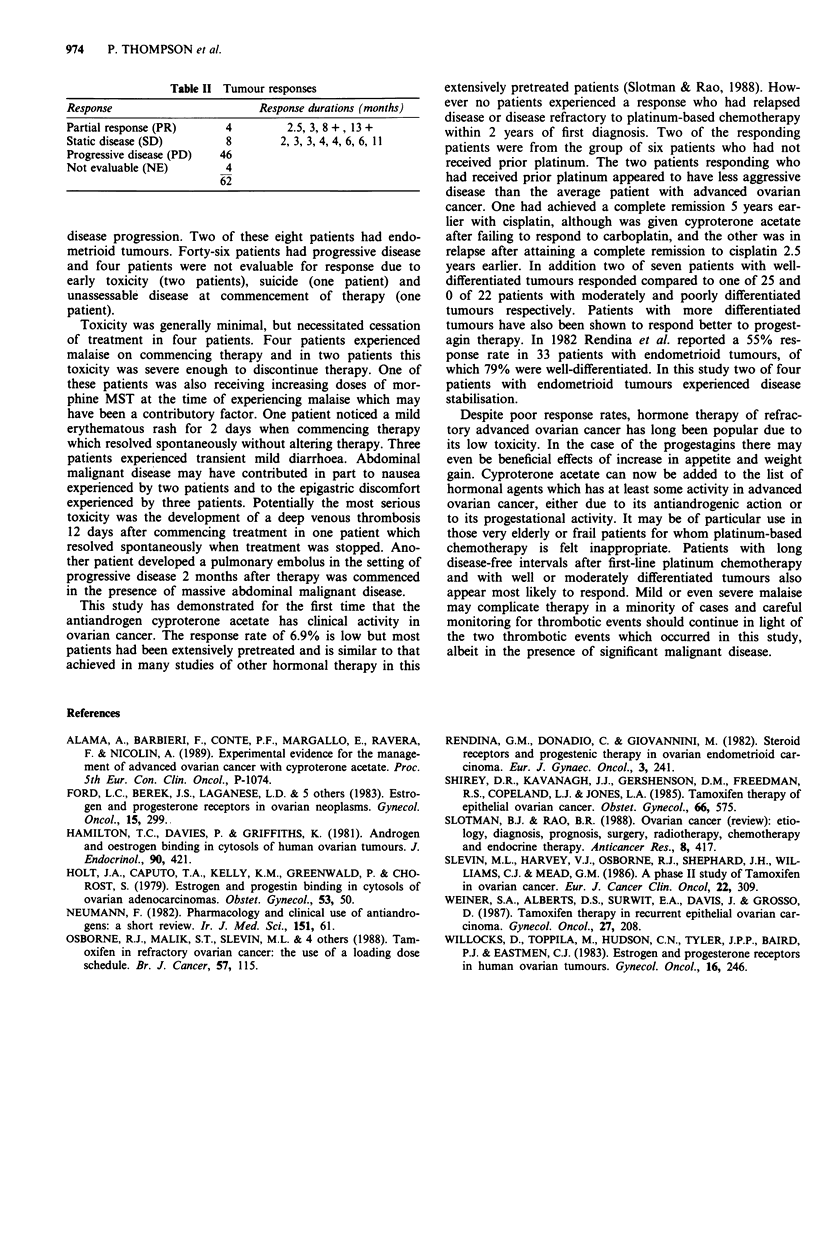# The clinical activity of cyproterone acetate in advanced ovarian carcinoma. A London Gynaecology Oncology Group Study.

**DOI:** 10.1038/bjc.1991.440

**Published:** 1991-11

**Authors:** P. Thompson, P. Wilson, R. Osborne, M. Slevin, F. Wiltshaw, P. Blake, P. Harper, R. Coleman, C. Williams, J. Sweetenham

**Affiliations:** Department of Medical Oncology, St Bartholomew Hospital, London, UK.


*Br. J. Cancer* (1991), **64**, 973–974 © Macmillan Press Ltd., 1991

SHORT COMMUNICATION

### The clinical activity of cyproterone acetate in advanced ovarian carcinoma. A London Gynaecology Oncology Group Study

P. Thompson[1], P. Wilson[1], R. Osborne[1], M. Slevin[1], E. Wiltshaw[2], P. Blake[2], P. Harper[3], R. Coleman[3], C. Williams[4], J. Sweetenham[4], A. Young[5] & R. Leonard[5]

*Departments of Medical Oncology, [1]St Bartholomew's and Homerton Hospitals, London; [2]Royal Marsden Hospital, Fulham, London; [3]Guy's Hospital, London; [4]Southampton General Hospital, Southampton; and [5]Western General Infirmary, Edinburgh, UK.*

The outlook for patients with advanced ovarian cancer who experience disease relapse following treatment with platinum-based chemotherapy is poor. As well as searching for more effective cytotoxic chemotherapy regimens recent attention has also focused on identifying those agents which have minimal toxicity but still retain clinical activity. In response to the discovery of oestrogen and progesterone receptors on ovarian cancer cells (Holt *et al.*, 1979; Rendina *et al.*, 1982; Ford *et al.*, 1983; Willocks *et al.*, 1983) hormonal therapy with either tamoxifen or progestagins has been undertaken by many investigators. However to date therapeutic results have been disappointing with response rates to tamoxifen ranging from 0% to 10% (Shirey *et al.*, 1985; Slevin *et al.*, 1986; Weiner *et al.*, 1987; Osborne *et al.*, 1988) and response rates to various progestagins ranging from 0% to 20% (Slotman & Rao, 1988).

Androgen receptors have now also been identified on ovarian cancer cells (Hamilton *et al.*, 1981) suggesting that therapy with androgen antagonists may have therapeutic potential. Cyproterone acetate is a steroidal antiandrogen which blocks the androgen receptor but in addition has potent progestational and antigonadotropic effects (Neumann, 1982). In 1989 Alma *et al.* reported that culture of an ovarian cancer cell line exhibiting androgen receptors with $10^{-5}$ M cyproterone acetate for 24 h resulted in the accumulation of 94% of cells in the $G_0/G_1$ phase of the cell cycle. The same investigators also reported the effect of cyproterone acetate 150 mg per day for 1 week in five patients with advanced ovarian cancer in relapse after several chemotherapy regimens including cisplatin. Tumour proliferative activity as assessed by the thymidine labelling index of malignant ascitic cells was assessed prior to and following the 1 week of therapy. All patients showed a reduction in thymidine labelling index with post treatment values ranging from 40% to 80% of pretreatment values suggesting that cyproterone may at least have some cytostatic effect *in vivo*. However to date there are no clinical studies reporting the outcome of long term therapy and the current study was designed to further assess the therapeutic potential of cyproterone acetate in refractory ovarian cancer patients.

Fifty-six patients with advanced ovarian cancer either refractory to or relapsing after platinum-based chemotherapy and with a life expectancy of greater than 2 months were treated. In addition six patients considered too frail for platinum-based chemotherapy were also treated. Standard WHO criteria were used to assess response and toxicity. Only patients with no radiological change in assessable disease were classified as static disease. Patients with overt or incipient bowel obstruction or with renal failure were excluded from the study. Table I demonstrates the patient and tumour characteristics.

Treatment consisted of continuous oral cyproterone acetate 100 mg three times daily. Treatment was only stopped for progression of disease or toxicity. The median duration of treatment was 10 weeks.

Details of tumour response and durations of response or static disease status are summarised in Table II. Fifty-eight patients are evaluable for response. Four patients (6.8%), all with serous or mucinous cystadenocarcinomas, experienced partial responses (PR) for 2.5, 3, 17 + and 18 + months. Two of the four responding patients had not received prior platinum-containing chemotherapy due to frailty. One of these received cyproterone acetate immediately after progressing on chlorambucil and the other had also progressed on chlorambucil having had a complete remission of disease on chlorambucil 3 years earlier. Of the other two responding patients one, who achieved a PR for 17 + months, was treated immediately after failing to respond to carboplatin having experienced a complete remission on cisplatin 5 years previously. The other, who achieved a PR for 18 + months, was in relapse 2.5 years after achieving a complete remission with cisplatin. In addition two of seven patients with well differentiated tumours responded compared to one of 25 and 0 of 22 patients with moderately and poorly differentiated tumours respectively. In the other responding patient tumour grade was unknown.

A further eight patients (13.8%) demonstrated static disease (SD) for 2 to 11 months before experiencing further

**Table I** Patient characteristics

| | |
|---|---|
| No. of patients | 62 |
| – prior platinum | 56 |
| – previously untreated (due to age or frailty) | 6 |
| Median age | 63 |
| Median no. previous chemotherapy regimens | 2 |
| Mean Karnofsky Performance Status | 83% |
| Stage at diagnosis (no. of patients): | |
| I | 1 |
| II | 6 |
| III | 38 |
| IV | 9 |
| Unknown | 8 |
| Histological grade (no. of patients): | |
| well differentiated | 7 |
| moderately differentiated | 25 |
| poorly differentiated | 22 |
| undifferentiated | 1 |
| unknown | 7 |
| Histological type (no. of patients): | |
| serous | 35 |
| mucinous | 6 |
| clear cell | 4 |
| endometrioid | 4 |
| unclassified adenocarcinoma | 9 |
| undifferentiated | 1 |
| unknown | 3 |

Correspondence: M.L. Slevin, ICRF Department of Medical Oncology, St Bartholomew's Hospital, West Smithfield, London EC1A 7BE, UK.
Received 8 August 1990; and in revised form 12 June 1991.

**Table II** Tumour responses

| Response | | Response durations (months) |
|---|---|---|
| Partial response (PR) | 4 | 2.5, 3, 8 +, 13 + |
| Static disease (SD) | 8 | 2, 3, 3, 4, 4, 6, 6, 11 |
| Progressive disease (PD) | 46 | |
| Not evaluable (NE) | 4 | |
| | 62 | |

disease progression. Two of these eight patients had endometrioid tumours. Forty-six patients had progressive disease and four patients were not evaluable for response due to early toxicity (two patients), suicide (one patient) and unassessable disease at commencement of therapy (one patient).

Toxicity was generally minimal, but necessitated cessation of treatment in four patients. Four patients experienced malaise on commencing therapy and in two patients this toxicity was severe enough to discontinue therapy. One of these patients was also receiving increasing doses of morphine MST at the time of experiencing malaise which may have been a contributory factor. One patient noticed a mild erythematous rash for 2 days when commencing therapy which resolved spontaneously without altering therapy. Three patients experienced transient mild diarrhoea. Abdominal malignant disease may have contributed in part to nausea experienced by two patients and to the epigastric discomfort experienced by three patients. Potentially the most serious toxicity was the development of a deep venous thrombosis 12 days after commencing treatment in one patient which resolved spontaneously when treatment was stopped. Another patient developed a pulmonary embolus in the setting of progressive disease 2 months after therapy was commenced in the presence of massive abdominal malignant disease.

This study has demonstrated for the first time that the antiandrogen cyproterone acetate has clinical activity in ovarian cancer. The response rate of 6.9% is low but most patients had been extensively pretreated and is similar to that achieved in many studies of other hormonal therapy in this extensively pretreated patients (Slotman & Rao, 1988). However no patients experienced a response who had relapsed disease or disease refractory to platinum-based chemotherapy within 2 years of first diagnosis. Two of the responding patients were from the group of six patients who had not received prior platinum. The two patients responding who had received prior platinum appeared to have less aggressive disease than the average patient with advanced ovarian cancer. One had achieved a complete remission 5 years earlier with cisplatin, although was given cyproterone acetate after failing to respond to carboplatin, and the other was in relapse after attaining a complete remission to cisplatin 2.5 years earlier. In addition two of seven patients with well-differentiated tumours responded compared to one of 25 and 0 of 22 patients with moderately and poorly differentiated tumours respectively. Patients with more differentiated tumours have also been shown to respond better to progestagin therapy. In 1982 Rendina *et al.* reported a 55% response rate in 33 patients with endometrioid tumours, of which 79% were well-differentiated. In this study two of four patients with endometrioid tumours experienced disease stabilisation.

Despite poor response rates, hormone therapy of refractory advanced ovarian cancer has long been popular due to its low toxicity. In the case of the progestagins there may even be beneficial effects of increase in appetite and weight gain. Cyproterone acetate can now be added to the list of hormonal agents which has at least some activity in advanced ovarian cancer, either due to its antiandrogenic action or to its progestational activity. It may be of particular use in those very elderly or frail patients for whom platinum-based chemotherapy is felt inappropriate. Patients with long disease-free intervals after first-line platinum chemotherapy and with well or moderately differentiated tumours also appear most likely to respond. Mild or even severe malaise may complicate therapy in a minority of cases and careful monitoring for thrombotic events should continue in light of the two thrombotic events which occurred in this study, albeit in the presence of significant malignant disease.

### References

ALAMA, A., BARBIERI, F., CONTE, P.F., MARGALLO, E., RAVERA, F. & NICOLIN, A. (1989). Experimental evidence for the management of advanced ovarian cancer with cyproterone acetate. *Proc. 5th Eur. Con. Clin. Oncol.*, P-1074.

FORD, L.C., BEREK, J.S., LAGANESE, L.D. & 5 others (1983). Estrogen and progesterone receptors in ovarian neoplasms. *Gynecol. Oncol.*, **15**, 299..

HAMILTON, T.C., DAVIES, P. & GRIFFITHS, K. (1981). Androgen and oestrogen binding in cytosols of human ovarian tumours. *J. Endocrinol.*, **90**, 421.

HOLT, J.A., CAPUTO, T.A., KELLY, K.M., GREENWALD, P. & CHOROST, S. (1979). Estrogen and progestin binding in cytosols of ovarian adenocarcinomas. *Obstet. Gynecol.*, **53**, 50.

NEUMANN, F. (1982). Pharmacology and clinical use of antiandrogens: a short review. *Ir. J. Med. Sci.*, **151**, 61.

OSBORNE, R.J., MALIK, S.T., SLEVIN, M.L. & 4 others (1988). Tamoxifen in refractory ovarian cancer: the use of a loading dose schedule. *Br. J. Cancer*, **57**, 115.

RENDINA, G.M., DONADIO, C. & GIOVANNINI, M. (1982). Steroid receptors and progestenic therapy in ovarian endometrioid carcinoma. *Eur. J. Gynaec. Oncol.*, **3**, 241.

SHIREY, D.R., KAVANAGH, J.J., GERSHENSON, D.M., FREEDMAN, R.S., COPELAND, L.J. & JONES, L.A. (1985). Tamoxifen therapy of epithelial ovarian cancer. *Obstet. Gynecol.*, **66**, 575.

SLOTMAN, B.J. & RAO, B.R. (1988). Ovarian cancer (review): etiology, diagnosis, prognosis, surgery, radiotherapy, chemotherapy and endocrine therapy. *Anticancer Res.*, **8**, 417.

SLEVIN, M.L., HARVEY, V.J., OSBORNE, R.J., SHEPHARD, J.H., WILLIAMS, C.J. & MEAD, G.M. (1986). A phase II study of Tamoxifen in ovarian cancer. *Eur. J. Cancer Clin. Oncol*, **22**, 309.

WEINER, S.A., ALBERTS, D.S., SURWIT, E.A., DAVIS, J. & GROSSO, D. (1987). Tamoxifen therapy in recurrent epithelial ovarian carcinoma. *Gynecol. Oncol.*, **27**, 208.

WILLOCKS, D., TOPPILA, M., HUDSON, C.N., TYLER, J.P.P., BAIRD, P.J. & EASTMEN, C.J. (1983). Estrogen and progesterone receptors in human ovarian tumours. *Gynecol. Oncol.*, **16**, 246.